# Machine learning for predicting neoadjuvant chemotherapy effectiveness using ultrasound radiomics features and routine clinical data of patients with breast cancer

**DOI:** 10.3389/fonc.2024.1485681

**Published:** 2025-01-14

**Authors:** Pu Zhou, Hongyan Qian, Pengfei Zhu, Jiangyuan Ben, Guifang Chen, Qiuyi Chen, Lingli Chen, Jia Chen, Ying He

**Affiliations:** ^1^ Cancer Research Center Nantong, Affiliated Tumor Hospital of Nantong University, and Medical School of Nantong University, Nantong, China; ^2^ Department of Ultrasound, Affiliated Tumor Hospital of Nantong University, Jiangsu, Nantong, China; ^3^ Department of Surgery, Affiliated Tumor Hospital of Nantong University, Nantong, China; ^4^ Department of Oncology Internal Medicine, Nantong Tumor Hospital, Affiliated Tumor Hospital of Nantong University, Nantong, China

**Keywords:** breast cancer, NAC, ultrasound radiomics features, pCR, GBM, SHAP

## Abstract

**Background:**

This study explores the clinical value of a machine learning (ML) model based on ultrasound radiomics features of primary foci, combined with clinicopathologic factors to predict the pathological complete response (pCR) of neoadjuvant chemotherapy (NAC) for patients with breast cancer (BC).

**Method:**

We retrospectively analyzed ultrasound images and clinical information from 231 participants with BC who received NAC. These patients were randomly assigned to training and validation cohorts. Tumor regions of interest (ROI) were delineated, and radiomics features were extracted. Z-score normalization, Pearson correlation analysis, and the least absolute shrinkage selection operator (LASSO) were utilized for further screening ultrasound radiomics and clinical features. Univariate and multivariate logistic regression analysis were performed to identify the CFs that were independently associated with pCR. We compared 10 ML models based on radiomics features: support vector machine (SVM), logistic regression (LR), random forest, extra trees (ET), naïve Bayes (NB), k-nearest neighbor (KNN), multilayer perceptron (MLP), gradient boosting ML (GBM), light GBM (LGBM), and adaptive boost (AB). Diagnostic performance was evaluated using the receiver operating characteristic (ROC) area under the curve (AUC), accuracy, sensitivity, and specificity, and the Rad score was calculated. Subsequently, construction of clinical predictive models and Rad score joint clinical predictive models using ML algorithms for optimal diagnostic performance. The diagnostic process of the ML model was visualized and analyzed using SHapley Additive exPlanation (SHAP).

**Results:**

Out of 231 participants with BC, 98 (42.42%) achieved pCR, and 133 (57.58%) did not. Twelve radiomics features were identified, with the GBM model demonstrating the best predictive performance (AUC of 0.851, accuracy of 0.75, sensitivity of 0.821, and specificity of 0.698). The clinical feature prediction model using the GBM algorithm had an AUC of 0.819 and an accuracy of 0.739. Combining the Rad score with clinical features in the GBM model resulted in superior predictive performance (AUC of 0.939 and an accuracy of 0.87). SHAP analysis indicated that participants with a high Rad score, PR-negative, ER-negative and human epidermal growth factor receptor-2 (HER-2) positive were more possibly to reach pCR. Based on the decision curve analysis, it was shown that the combined model of GBM provided higher clinical benefits.

**Conclusion:**

The GBM model based on ultrasound radiomics features and routine clinical date of BC patients had high performance in predicting pCR. SHAP analysis provided a clear explanation for the prediction results of the GBM model, revealing that patients with a high Rad score, PR-negative status, ER-negative status and HER-2-positive status are more likely to achieve pCR.

## 1 Introduction

BC is the most common malignant tumor among women (1). Highly invasive BC is challenging to treat and is characterized by a high recurrence rate and poor prognosis (2, 3). While surgery (4) remains the primary treatment for BC, some patients are not suitable for direct surgery due to large tumor lesions, extensive metastases, or a strong preference for breast preservation. Neoadjuvant chemotherapy (NAC) is administered to reduce the clinical stage, improve the likelihood of breast-preservation, and decrease the need for axillary surgery (5, 6). Thus, assessing the efficacy of NAC is crucial for determining the subsequent individualized treatment plan. Current methods for evaluating the effectiveness of NAC primarily include pathological and clinical assessments. Among clinical assessment methods, ultrasound is more frequently utilized than magnetic resonance imaging (MRI) and mammography (7). However, imaging techniques like ultrasound and MRI, as well as non-imaging methods such as pathological evaluation, more often fall short of characterizing the therapeutic effectiveness of NAC by rule and line. While pathological assessment, though the gold standard for efficacy evaluation, suffers from delayed results (8). Pathological complete remission (pCR) following NAC is strongly associated with favorable outcomes (6), and is a key metric for evaluating the effectiveness of NAC. pCR can serve as an early surrogate endpoint for predicting improved disease-free survival (DFS) and overall survival (OS) in patients after NAC (9). Therefore, early prediction of the systemic response of BC to NAC is clinically significant, enabling clinicians to adjust treatment plans promptly, minimize unnecessary chemotherapy side effects, enhance pCR rates, and improve patient prognosis.

With the rapid advancement of machine learning (ML) algorithms and their applications in clinical cancer research, cancer prediction performance has significantly improved (10, 11). ML is increasingly used in the medical field for predicting outcomes, diagnosing conditions, and guiding treatments (12). However, the logical thinking and complex calculation of various ML algorithms can differ (13), leading to variations in clinical applications. For instance, a study (14) comparing the prediction performance of different ML algorithms for BC recurrence found that the adaptive boost (AB) algorithm gained optimal performance (AUC of 0.987). ML offers advantages over traditional methods in the area of precision and velocity, and it can recognize new predictive features and spatial patterns that may be missed by human analysis (15, 16). By extracting valuable clinical information from large datasets, ML helps make informed clinical decisions. The SHAP method provides both holistic and localized explainability. It explains model predictions by attributing them to the contributed value from each of the input features, known as the Shapley value. Comparison with other interpretative methods, SHAP offers a clearer visualization of the prediction process for complex ML models. Several researchers have incorporated Explainable Artificial Intelligence (XAI) techniques to analyze the efficacy of chemotherapy for targeted cancers (17). Zhang (18) et al. developed an ML model for accurately predicting the probability of obtaining a pCR after neoadjuvant chemotherapy (NAC) in patients with locally advanced breast cancer (LABC). Furthermore, the ML model was visualized and analyzed using SHAP technology. However, there may be some limitations in discussing only the influence of US images or clinical factors on pCR. Therefore, this study aimed to develop an ML model that integrates BC ultrasound and radiomics data with clinical factors to predict the pCR following NAC. The predictive results of the ML model were interpreted visually by using SHAP. The study seeks to guide clinicians in developing personalized diagnosis and treatment plans for patients with BC. The flowchart of RFs and CFs extraction and models establishment is shown in **Figure 2**.

## 2 Materials and methods

### 2.1 Patients

**Figure 1:**
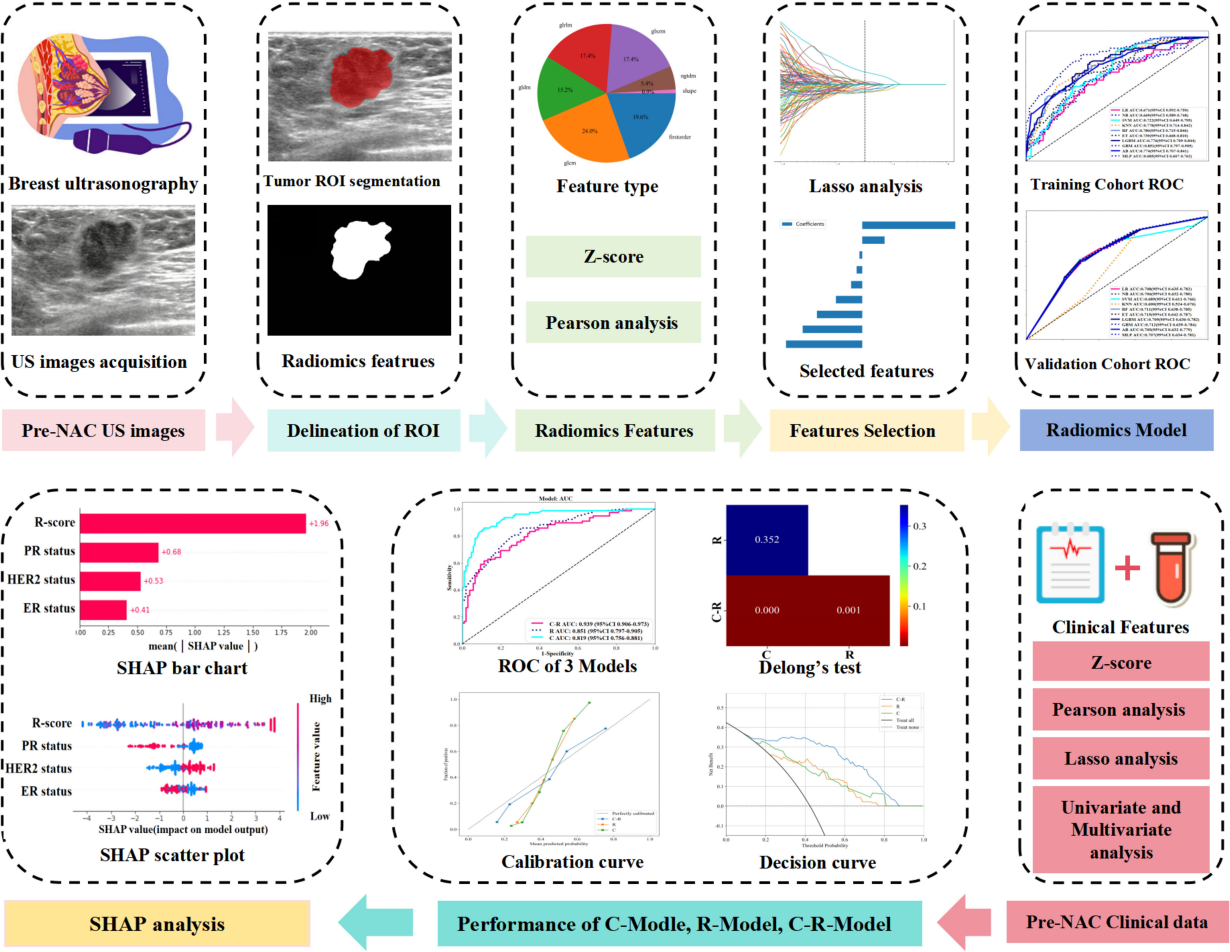
Flowchart of radiomics features and clinical features extraction, model establishment and analysis.

The study included patients diagnosed with BC between December 2014 and September 2023 who underwent NAC at the Affiliated Tumor Hospital of Nantong University. The diagnosis of BC was confirmed through surgical and pathological means. The inclusion criteria were listed as following conditions: (a) patients with pathologic results of pCR or non-pCR after NAC and surgery; (b) patients treated with a full course of NAC; and (c) patients who provided preoperative breast ultrasound examination and puncture biopsy results. The exclusion criteria were listed as following conditions: (a) patients with unavailable and incomplete pathological results; (b) patients who did not receive a full course of NAC; (c) patients with inadequate ultrasound image quality; (d) patients with bilateral breast tumors and unilateral multifocal carcinomas. **Figure 1** showed a patient enrollment flow chart. The study adhered to the Declaration of Helsinki and was approved by the Ethics Committee of the Affiliated Tumor Hospital of Nantong University (No. LW2024024). Written informed consent was obtained from each patient. The final enrolled 231 patients were randomly assigned to the training cohort (n = 185) and the validation cohort (n = 46) (**Table 1**).

**Figure 2:**
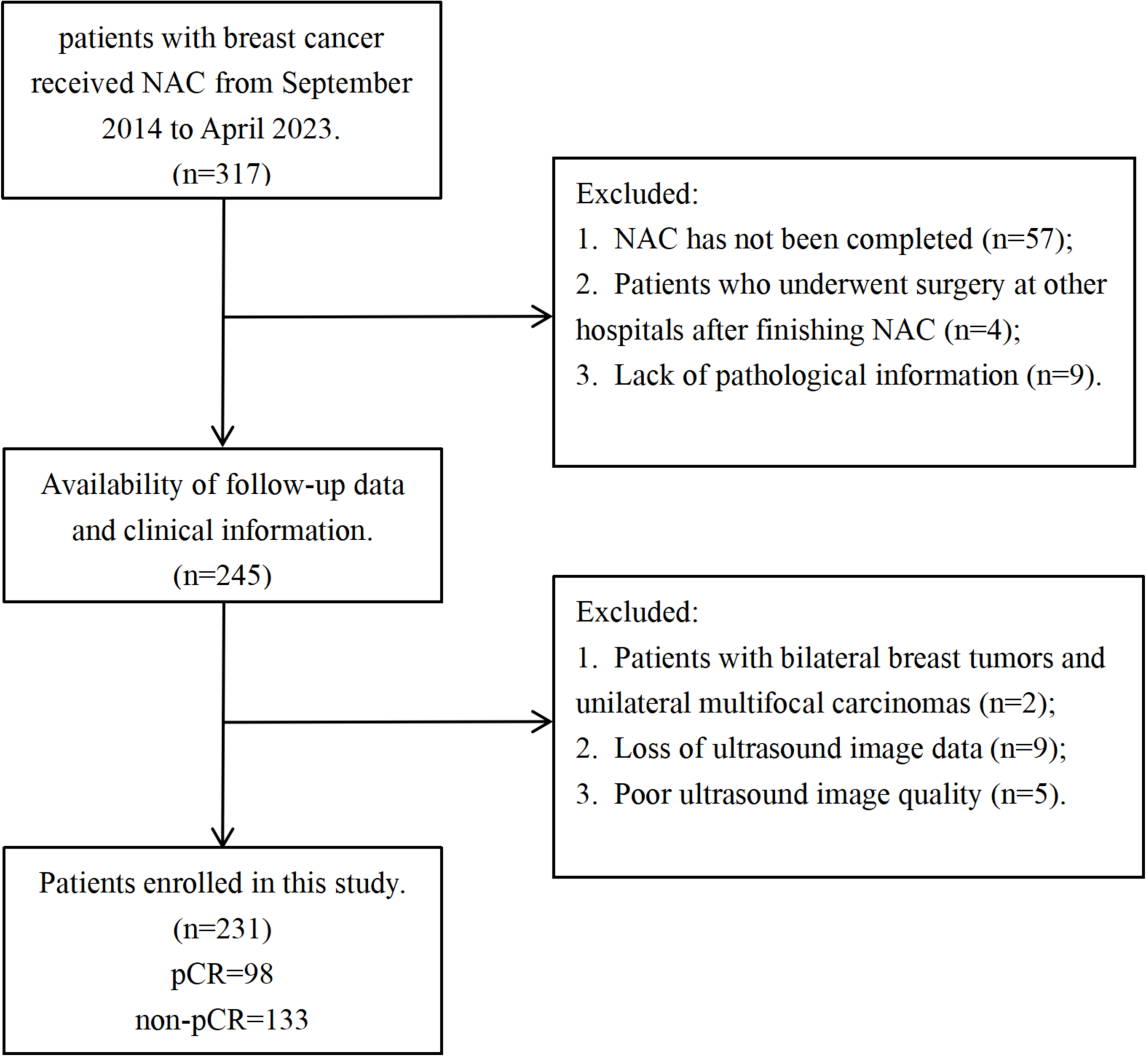
Flowchart of patient enrollment.

**Table 1:** Baseline characteristics of the participants.

Characteristics		Training Cohort (N=185)	Validation Cohort (N=46)	P-value
Age, Mean (SD), years		54.49 (± 9.539)	56.13 (± 9.570)	0.300
Menopause	yes	104 (56.2%)	23 (50.0%)	0.450
	no	81 (43.8%)	23 (50.0%)	
Breastfeeding history	yes	174 (94.1%)	44 (95.7%)	0.304
	no	11 (5.9%)	2 (4.4%)	
Family history of breast cancer	yes	9 (4.8%)	0	0.128
	no	176 (95.2%)	46 (100%)	
Basic diseases	yes	35 (18.9%)	5 (10.9)	0.198
	no	150 (81.1%)	41 (89.11%)	
Chemotherapy	AC-T	76 (41.1%)	24 (52.2%)	0.490
	TCbHP	42 (22.6%)	8 (17.4%)	
	AT	19 (10.2%)	2 (4.3%)	
	TP	14 (7.5%)	4 (8.7%)	
	AC	11 (5.9%)	3 (6.5%)	
	TC	23 (12.4%)	5 (10.9%)	
Pathological type	Invasive ductal carcinoma	157 (84.9%)	37 (80.4%)	0.444
	Invasive lobular carcinoma	8 (4.3%)	2 (4.3%)	
	Others	20 (10.8%)	7 (15.3%)	
Clinical T Stage	T1-T2	120 (64.9%)	33 (71.7%)	0.579
	T3-T4	65 (35.1%)	13 (28.3%)	
Clinical N Stage	N0-N1	136 (75.1%)	35 (76.1%)	1.000
	N2-N3	49 (24.9%)	11 (23.9%)	
Molecular subtype	Luminal A-like	16 (8.6%)	5 (10.9%)	0.673
	Luminal B-like	83 (44.9%)	19 (41.3%)	
	HER2-enriched	66 (35.7%)	15 (32.6%)	
	Triple negative	20 (10.8%)	7 (15.2%)	
ER status	negative	87 (47.0%)	22 (47.8%)	0.718
	positive	98 (53.0%)	24 (52.2%)	
PR status	negative	123 (66.5%)	31 (67.4%)	0.908
	positive	62 (33.5%)	15 (32.6%)	
HER2 status	negative	86 (46.5%)	23 (50.0%)	0.871
	positive	99 (53.5%)	23 (50.0%)	
ki67 status	<14%	40 (21.6%)	9 (19.6%)	0.809
	≥14%	145 (78.4%)	37 (80.4%)	
Location	Left	98 (52.9%)	26 (56.5%)	0.550
	Right	87 (47.1%)	20 (43.5%)	
CEA, Mean (SD), ng/ml		9.08 (± 2.864)	137.96 (± 133.368)	0.341
CA125, Mean (SD), U/ml		21.19 (± 1.868)	24.08 (± 4.290)	0.505
CA153, Mean (SD), U/ml		21.66 (± 1.355)	29.54 (± 5.649)	0.183
CA50, Mean (SD), U/ml		10.69 (± 1.637)	8.85 (± 1.134)	0.580
Miller-Payne	pCR	78 (42.2%)	20 (43.4%)	0.982
	non-pCR	107 (57.8%)	26 (56.6%)	

SD, standard deviation; AC-T, adriamycin with cyclophosphamide plus docetaxel; TCbHP, docetaxel with carboplatin and trastuzumab and pertuzumab; AT, adriamycin with docetaxel; TP, docetaxel with pertuzumab; AC, adriamycin with cyclophosphamide; TC, docetaxel with cyclophosphamide; ER, estrogen receptor; PR, progesterone receptor; HER-2, human epidermal growth factor receptor-2; CEA, carcinoembryonic antigen; CA153, carbohydrate antigen 153; CA125,carbohydrate antigen 125; CA50, carbohydrate antigen 50; pCR, pathological complete response.

### 2.2 Effectiveness and pathological assessment of NAC

The National Comprehensive Cancer Network (NCCN) guidelines adhered to guide treatment regimens and schedules for BC patients. The NAC for BC included anthracyclines (doxorubicin or epirubicin) either in combination with or followed by paclitaxel or docetaxel (19). The 231 participants were subjected to postoperative histopathology to evaluate their responsiveness to NAC. The criteria for pCR were defined as the absence of residual invasive carcinoma in the specimen (with or without residual ductal carcinoma *in situ*) and the absence of lymph node involvement in the ipsilateral anterior sentinel lymph nodes or axillary lymph nodes.

### 2.3 Clinical parameter

Clinical parameter comprised the patient’s age, menopausal status, history of breastfeeding, family history of cancer, and underlying diseases. Tumor-related information encompassed tumor pathology types (such as invasive ductal carcinoma, invasive lobular carcinoma, and others), molecular subtypes (such as luminal A-like, luminal B-like, human epidermal growth factor receptor-2 [HER-2] enriched, and triple negative), and tumor, node, and metastasis (TNM) stages (T-stage [1–4], N-stage [0–3]). Additional data included estrogen receptor (ER) status, progesterone receptor (PR) status, HER-2 status, Ki-67 expression (<20% or ≥20%), and the primary tumor location (left, right, or bilateral). Tumor biomarkers such as carcinoembryonic antigen (CEA), carbohydrate antigen 153 (CA153), carbohydrate antigen 125 (CA125), and carbohydrate antigen 50 (CA50) were also recorded. The TNM staging followed the 2017 American Joint Committee on Cancer (AJCC) eighth edition criteria for BC (**Table 1**).

### 2.4 Ultrasonography

Ultrasonography was conducted using the GE Logic E9 and Philips EPIQ7 diagnostic ultrasound machines. Four highly experienced doctors (with more than ten years’ experience in breast ultrasound) performed preoperative breast ultrasound. For 231 participants, we analyzed the images with the maximum diameter. The reader 1 and reader 2, each with at least ten years of experience in breast ultrasound and unaware of the pathologic results, segmented the region of interest (ROI) in the ultrasound images using Itk-Snap (version 3.8.0). One month later, the reader 3, with nine years of breast ultrasound interpretation experience, delineated 55 random patients’ ultrasound images. The interclass correlation coefficients (ICC) were used to evaluate the consistency of extracted feature between observers. ICC values are categorized as follows: <0.40 was considered “poor,” 0.40 to 0.59 was “fair,” 0.60 to 0.74 was “good,” and 0.74 to 1.00 was “excellent”.

### 2.5 Radiomics features extraction

We used the PyRadiomics open-source tool (available at: https://www.example.com/en/latest/index.html) to extract radiomics features (RFs) from the images. A full seven categories of features were extracted: (1) first-order; (2) gray-level co-occurrence matrix (GLCM); (3) gray-level dependence matrix (GLDM); (4) gray-level run-length matrix (GLRLM); (5) gray-level size-zone matrix (GLSZM); (6) neighboring gray-tone difference matrix (NGTDM); and (7) SHAPE features. These RFs were obtained from the pre-treatment ultrasound images before NAC.

### 2.6 Screening and validation of ML models

Before feature selection, the threshold value of ICC was greater than 0.75, which could ensure the repeatability and stability of the features. All ultrasound RFs and clinical features (CFs) extracted from the images were normalized using the Z-score method, followed by Pearson correlation analysis. The least absolute shrinkage selection operator (LASSO) was then applied to further filtrate the RFs and CFs, selecting those with the highest correlation based on the least squares error criterion. Univariate and multivariate logistic regression analysis were performed to identify the CFs that were independently associated with pCR. Subsequently, we compared 10 ML models based on RFs: support vector machine (SVM), logistic regression (LR), random forest (RF), extreme random trees (ET), naïve Bayes (NB), k-nearest neighbor (KNN), multilayer perceptron (MLP), gradient boosting ML (GBM), light GBM (LGBM), and AB. The diagnostic performance of these models was optimized using a mesh finding method to avoid overfitting.

The predictive performance of 10 ML models was comprehensively evaluated using AUC, accuracy, sensitivity, specificity, positive predictive value (PPV), and negative predictive value (NPV). Rad scores were calculated under each algorithm. We compared the radiomics model (R-model), the clinical feature model (C-model), and the combined Rad score and clinical feature model (C-R-model) using DeLong’s test. We use calibration curves to evaluate the calibration of predictive models, and decision curve analysis (DCA) to compute and contrast the net benefits of the training and validation cohorts under different threshold probabilities in order to evaluate the clinical value of three models.

### 2.7 Visualizing ML models

SHAP quantified the importance of each feature by calculating its contribution value, indicating whether its impact was positive or negative (20). This approach facilitated the analysis of the significance of each feature, thereby enhancing the clinical application of ML models.

### 2.8 Statistical analysis

Python (version 3.7), R (version 4.2.0), and IBM SPSS Statistics for Windows (version 25.0; IBM Corp., Armonk, NY, USA) were used to conduct statistical analyses. Normally distributed continuous variables were compared using the independent sample t-test, while categorical variables were assessed using the chi-square test. The performance of each model was evaluated using Z-scores, Pearson correlation analysis, LASSO screening of clinical features and RFs, and ROC curves. AUC was calculated.

## 3 Results

### 3.1 Clinicopathologic characteristics in participants

The 231 participants participated in this study. The flowchart of patient enrollment is shown in **Figure 1**. There were no statistically significant differences between the training and validation groups in terms of age, menopausal status, history of breastfeeding, family history of cancer, underlying disease, tumor pathology type, tumor molecular subtypes, TNM stage (T stage [1–4], N stage [0–3]), ER status, PR status, HER-2 status, Ki-67 expression, tumor location, CEA levels, CA153, CA125, CA50 levels, pCR, or non-pCR (**Table 1**).

### 3.2 Screening of RFs and R-model construction

**Figure 3:**
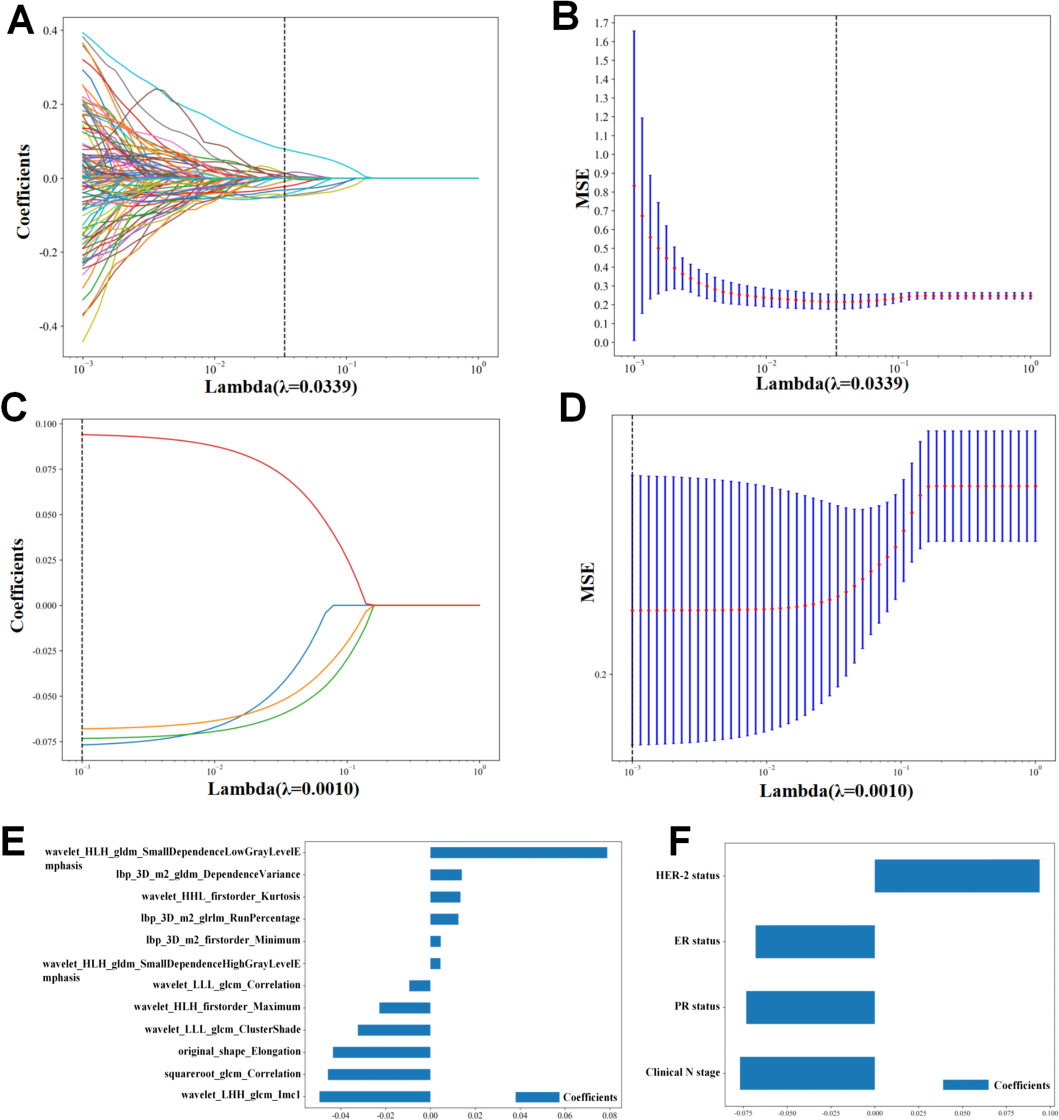
Radiomics feature and clinical feature extraction. **(A, B)** Lasso analysis of radiomics features. **(E)** Radiomics features’s coefficients. **(C, D)** Lasso analysis of clinical features. **(F)** Clinical features’s coefficients.

From the RF extraction, a total of 1,562 RFs were screened, including FIRSTORDER (16.8%), GLCM (22.4%), GLDM (13.1%), GLRLM (15%), GLSZM (15%), NGTDM (4.7%), and SHAPE (13.1%). Before selection, 1,064 features had an ICC of >0.75, ensuring their reproducibility. After applying Z-score normalization, Pearson correlation analysis, and LASSO regression analysis (**Figures 3A, B**), the results indicated that the R-model could be obtained with λ = 0.0168. Based on the screening of 12 RFs (**Figure 3E**), the Rad score formula was:

the Rad score = 0.4242424242424243

+ 0.004568 * lbp_3D_m2_firstorder_minimum

+ 0.014004 * lbp_3D_m2_gldm_DependenceVariance

+ 0.012488 * lbp_3D_m2_glrlm_RunPercentage

– 0.043531 * original_shape_Elongation

– 0.045768 * squareroot_glcm_Correlation

+ 0.013394 * wavelet_HHL_firstorder_Kurtosis

– 0.022782 * wavelet_HLH_firstorder_maximum

+ 0.078863 * wavelet_HLH_gldm_SmallDependenceHighGrayLevelEmphasis

– 0.049518 * wavelet_LHH_glcm_Imc1

+0.004476 * wavelet_LLH_gldm_SmallDependenceLowGrayLevelEmphasis

– 0.032368 * wavelet_LLL_glcm_ClusterShade

– 0.009410 * wavelet_LLL_glcm_Correlation

**Figure 4:**
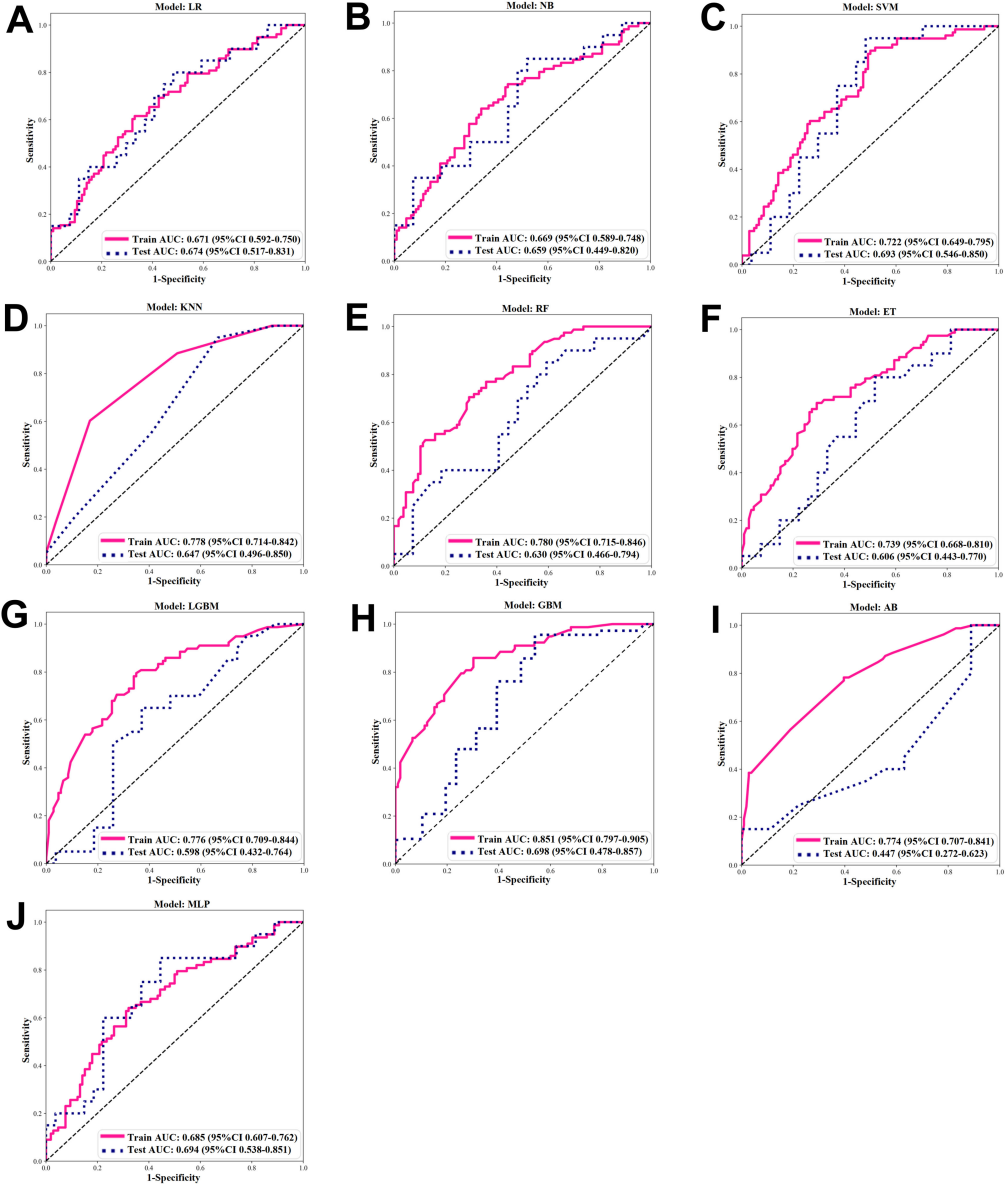
ROC of ML algorithms. **(A-J)** ROC under 10 ML algorithms in the Training and Validation Cohort. **(A)** Logistic regression (LR). **(B)** Naïve Bayes (NB). **(C)** Support vector machine (SVM). **(D)** K-nearest neighbor (KNN). **(E)** Random forest (RF). **(F)** extra trees (ET). **(G)** Light GBM (LGBM). **(H)** Gradient boosting ML (GBM). **(I)** Adaptive boost (AB). **(J)** Multilayer perceptron (MLP).

After comparing the ROC curves of 10 ML models of LR, NB, SVM, KNN, RF, ET, LGBM, GBM, AB, and MLP, the GBM model demonstrated the optimal predictive performance with an AUC of 0.851 and accuracy of 0.750 (**Figure 4**). Its sensitivity, specificity, PPV, and NPV were also superior to those of other algorithms in the training and validation cohorts (**Tables 2**, **3**).

**Table 2:** Screening evaluation metrics for machine learning algorithms using 10-fold cross-validation in the training cohort.

	LR	NB	SVM	KNN	RF	ET	LGBM	GBM	AB	MLP
AUC	0.671	0.669	0.722	0.778	0.780	0.739	0.776	0.851	0.774	0.685
Accuracy	0.641	0.647	0.663	0.630	0.701	0.696	0.707	0.750	0.674	0.658
Sensitivity	0.603	0.628	0.885	0.537	0.692	0.679	0.782	0.821	0.769	0.628
Specificity	0.670	0.660	0.500	0.593	0.708	0.708	0.651	0.698	0.604	0.679
PPV	0.573	0.576	0.566	0.778	0.635	0.635	0.622	0.667	0.588	0.590
NPV	0.696	0.707	0.855	0.614	0.758	0.758	0.802	0.841	0.780	0.713

**Table 3:** Screening evaluation metrics for machine learning algorithms using 10-fold cross-validation in the validation cohort.

	LR	NB	SVM	KNN	RF	ET	LGBM	GBM	AB	MLP
AUC	0.674	0.659	0.693	0.647	0.630	0.606	0.598	0.698	0.447	0.694
Accuracy	0.617	0.617	0.723	0.574	0.574	0.596	0.617	0.681	0.617	0.660
Sensitivity	0.750	0.800	0.750	0.550	0.800	0.750	0.600	0.900	0.650	0.800
Specificity	0.519	0.481	0.704	0.593	0.407	0.481	0.630	0.519	0.852	0.556
PPV	0.536	0.533	0.652	0.500	0.500	0.517	0.545	0.581	0.765	0.571
NPV	0.737	0.765	0.792	0.640	0.733	0.722	0.680	0.875	0.767	0.789

### 3.3 Screening of clinical features and C-model construction

Following Z-score normalization, Pearson correlation analysis, LASSO regression analysis (**Figures 3C, D**), Four features were filtered out (**Figure 3F**). Univariate and multivariate logistic regression analysis were performed to identify the CFs that were independently associated with pCR, three CFs were selected: HER-2 status, ER status, PR status (**Table 4**). The C-model was constructed with these features, and the optimal λ value was found to be 0.0043. We used the GBM algorithm for further analysis and developed the C-model based on the three selected clinical features. The results indicated that the C-model had an AUC of 0.819and an accuracy of 0.739 (**Table 5**).

**Table 4:** Univariate and multivariate analysis of clinical features according to the pCR.

Clinical features	Univariate		Multivariate	
	OR (95% CI)	P-value	OR (95% CI)	P-value
Clinical N Stage	0.463 (0.228-0.94)	0.033		
ER status	0.296 (0.161-0.545)	0.001	1.332 (0.382-4.64)	0.0113
PR status	3.29 (1.666-6.496)	0.001	2.173 (1.006-4.694)	0.048
HER2 status	4.201 (2.23-7.912)	0.001	3.228 (1.532-6.799)	0.002

**Table 5:** Performance comparison of C Model, R Model, C-R Model.

	Training Cohort			Validation Cohort		
	C-Model	R-Model	C-R-Model	C-Model	R-Model	C-R-Model
AUC	0.819	0.851	0.939	0.732	0.698	0.863
Accuracy	0.739	0.750	0.870	0.638	0.681	0.831
Sensitivity	0.628	0.821	0.846	0.900	0.900	0.750
Specificity	0.821	0.698	0.887	0.444	0.519	0.767
Precision	0.721	0.667	0.846	0.545	0.581	0.695
Recall	0.628	0.821	0.846	0.900	0.900	0.850
F1-score	0.671	0.736	0.846	0.679	0.706	0.782

### 3.4 Validation and clinical valuation of C-R-model

**Figure 5:**
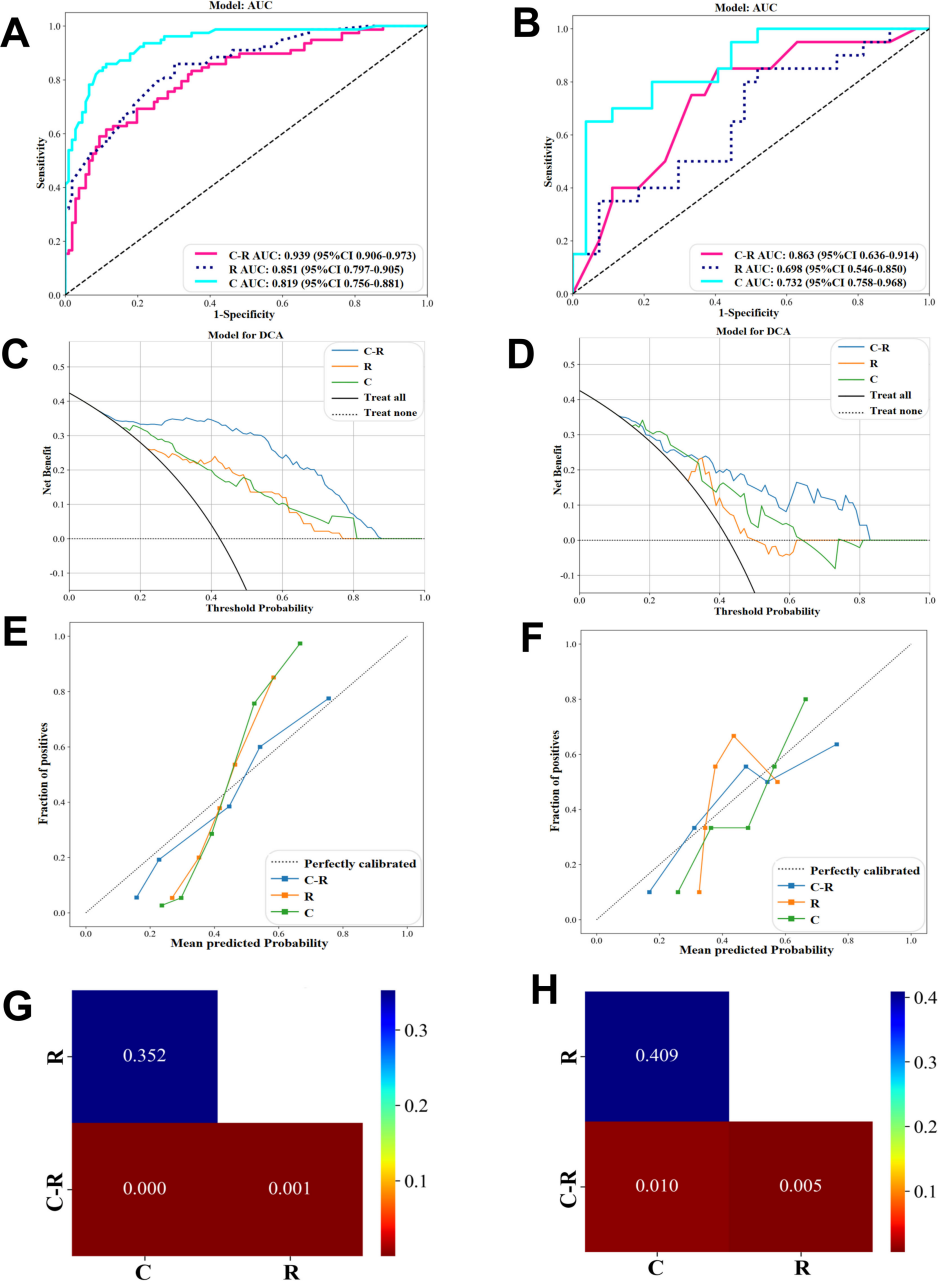
Performance comparison of C-Model, R-Model, C-R-Model in GBM algorithms. **(A, B)** ROC in the training and validation cohort. **(C, D)** DCA in the training and validation cohort. **(E, F)** Calibration curve in the training and validation cohort. **(G)** Cohort train Delong and **(H)** Cohort validation Delong.

Using the GBM algorithm (**Figures 5A, B**), we combined the three selected CFs with the Rad score to construct a C-R-model, which was then compared with the C-model and R-model. The C-R-model demonstrated the optimal predictive performance, with an AUC of 0.939 and accuracy of 0.870, outperforming both the C-model and R-model in terms of diagnostic accuracy in both the training and validation cohorts. What’s more, the C-R-model resulted in superior predictive performance comparing with existing pCR prediction models. A visual nomogram (**Supplementary Figure 1A**) was developed using Rad-score combined with PR status, ER status, HER-2 status. The nomogram yielded an AUC of 0.926 in the training set (**Supplementary Figure 1B**). In the validation set (**Supplementary Figure 1C**), the AUC was 0.832. We utilized DCA to compare the clinical benefits of the C-R model with those of the C-model and R-model. Overall, the C-R-model, based on the GBM algorithm, demonstrated superior clinical benefits (**Figures 5C, D**). Additionally, the calibration curves showed that the C-R-model outperformed both the C-model and R-model in calibration performance, as evidenced in the training and validation cohorts (**Figures 5E, F**). We used DeLong’s test (**Figures 5G, H**) to statistically compare the three models. The C-R-model showed a statistically significant improvement over the C-model and the R-model, while no significant difference was found between the C-model and R-model.

### 3.5 SHAP analysis

**Figure 6:**
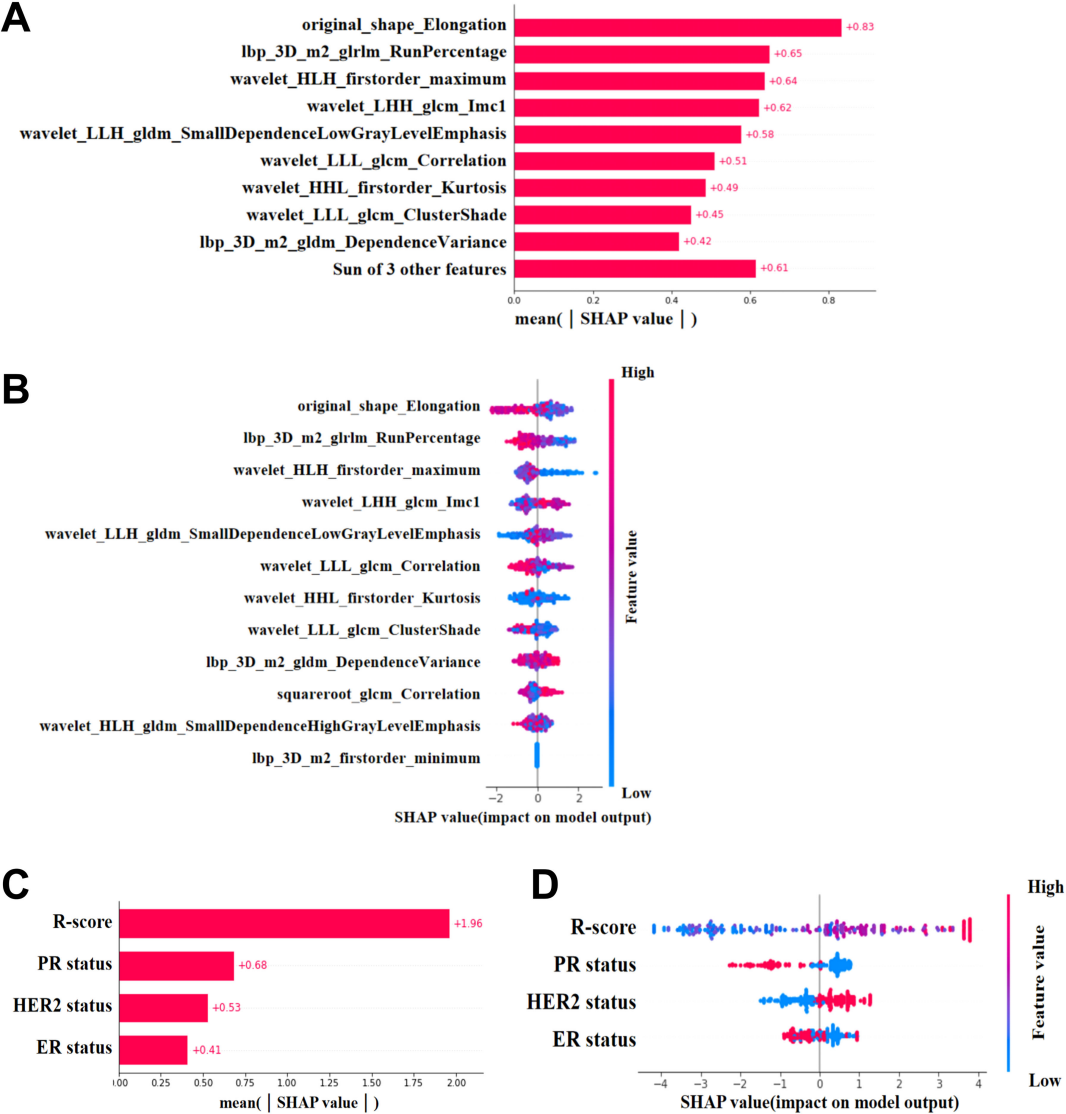
SHAP. **(A, C)** SHAP(R-Model and C-R-Model) bars show the weights of the most important features of the model. **(B, D)** SHAP(R-Model and C-R-Model) scatter plot shows the positive or negative impact of each characteristic on the predicted probability in red and blue.

**Figure 7:**
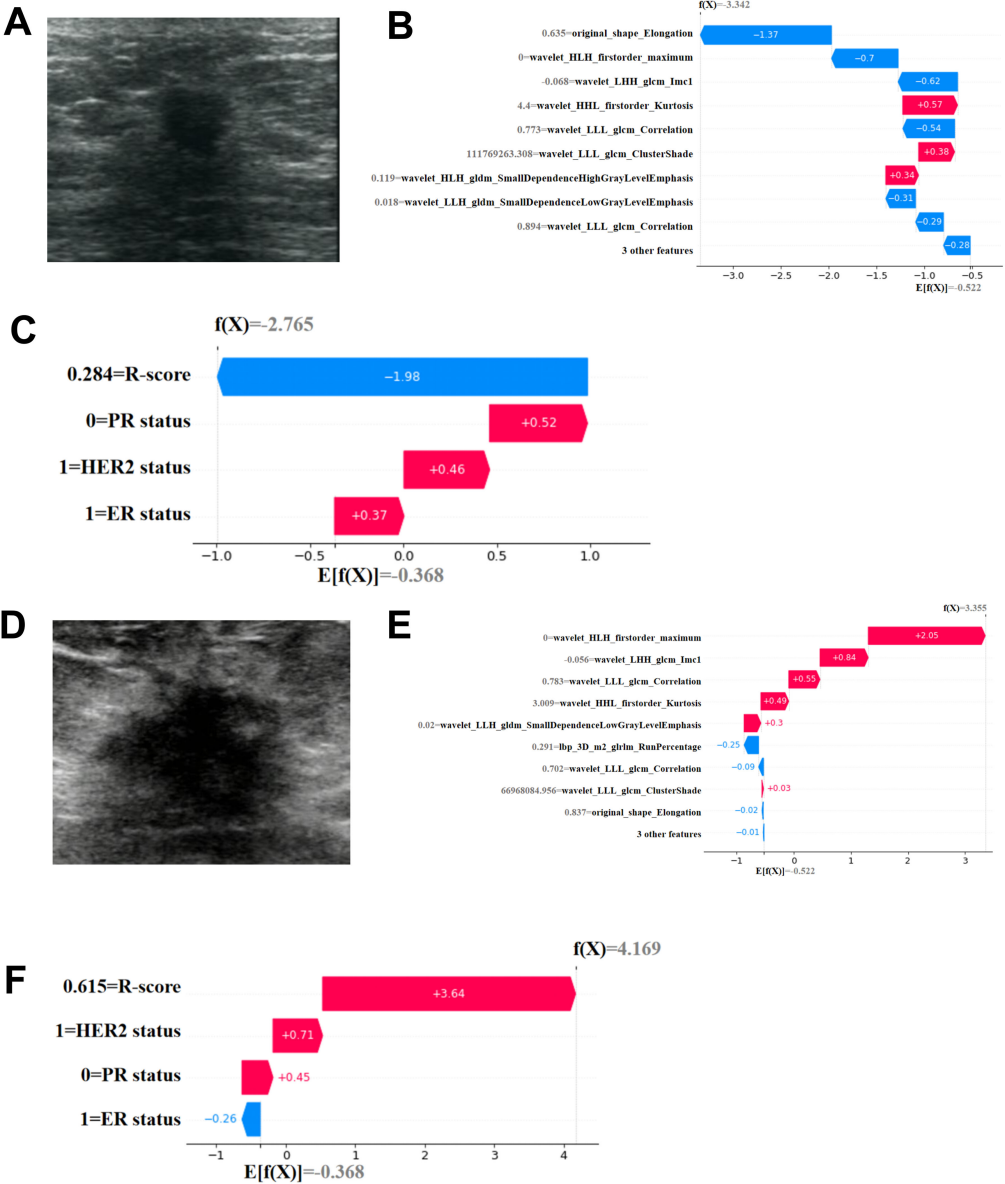
Individual visualization of the mode through SHAP. **(A-C)** The data comes from a female patient, 53 years old. **(A)** B-US image of lesion. For this patient, the predicted outcome of SHAP(R-Model) waterfall plot **(B)** was -3.342 (baseline: -0.522) and he predicted outcome of SHAP(C-R-Model) waterfall plot **(C)** was -2.765 (baseline: -0.368), with a predicted outcome of non pCR. The final pathological result was Miller Payne grade 2, which did not achieve complete pathological remission. **(D-F)** The data comes from a female patient, 67 years old. **(D)** B-US image of lesion. For this patient, the predicted outcome of SHAP(R-Model) waterfall plot **(E)** was 3.355 (baseline: -0.522) and he predicted outcome of SHAP(C-R-Model) waterfall plot **(F)** was 4.169 (baseline: -0.368), with a predicted pCR. The final pathological result was Miller Payne grade 5, achieving complete pathological remission.

This study used SHAP to visualize the results of the GBM model (C-R-model and R-model). The SHAP(R-model) bar chart (**Figure 6A**) illustrates the importance of the 12 most significant RFs, where the y-axis represents the features sorted according to the importance rankings from top to bottom, the original_shape_Elongation had the greatest impact. Meanwhile, the SHAP(C-R-model) bar chart (**Figure 6C**) illustrates the importance of the four most significant features: Rad score, PR status, ER status, HER-2 status. The Rad score had the greatest impact on predicting pCR after NAC in BC, followed by PR status, ER status, HER-2 status. The SHAP(R-model and C-R-model) scatter plot (**Figures 6B, D**) visualizes the positive or negative impact of each feature on the predicted probability, with red indicating a positive impact and blue indicating a negative impact. According to **Figure 6D**, patients with a high Rad score, PR-negative status, ER-negative status, and HER-2 positive status were more possibly to reach pCR. Further visualizations of the model using SHAP waterfall chart are shown in **Figure 7**. Although the waterfall charts of SHAP(C-R-model) and SHAP(R-model) both accurately predicted the sample, SHAP(C-R-model) (F1 0.81) was more stable than SHAP(R-model) (F1 0.58).

## 4 Discussion

This study retrospectively analyzed the ultrasound imaging, histology, and clinical characteristics of 231 participants with BC, evaluating 10 common ML algorithms. We found that the R-model under the GBM algorithm exhibited the best overall diagnostic performance. Based on the GBM algorithm, we established the C-model and C-R-model to predict the pCR in patients with BC undergoing NAC before treatment. Compared to the R-model and C-model, the C-R-model (AUC, 0.939; accuracy, 0.870) demonstrated superior predictive accuracy and clinical utility for assessing pCR. Key features for predicting pCR included the Rad score, PR status, ER status, and HER-2 status. SHAP analysis provided a clear explanation for the prediction results of the GBM model, revealing that participants with a high Rad score, PR-negative status, ER-negative status, HER-2-positive status are more likely to achieve pCR.

### 4.1 Prediction performance of CFs and RFs

Over the past decade, individualized treatment for patients with BC undergoing NAC has been a major research focus, with up to 60% of participants achieving pCR (21). Previous studies have identified hormone receptor and HER-2 status as crucial clinical predictors of treatment response (22). The biopsy identifies important factors such as hormone receptor (ER and PR) and HER-2, which contributed insights into treatment options and prognosis (23). Additionally, a single-center study (24) employing vacuum assisted biopsy (VAB) after NAC found no ipsilateral recurrences during a 26.4-month follow-up in patients who met specific criteria (CT1-2, clinical N stage [0–1], triple negative or HER-2 positive, with residual lesions on imaging following NAC, and tumors ≤2 cm). Park et al. (25) revealed that ER-negative status should be considered a prognostic factor of tailored NAC based on the status of molecular subtypes in breast cancer. Yao et al. (26) found The RF-based combined peritumoral intratumoral ultrasound radiomics signatures (P-IURS) model of the HER-2-positive status subtype improved the efficacy to a maximum AUC. Wang et al. uncovered (27) ER-negative patients had a significantly higher pCR rate: 36% (23/64) ER-negative patients achieving pCR while only 2% (3/125) for ER-positive patients, as with ER, PR-negative patients also had a better chance for reaching a pCR (34%, 25/74) than the positive ones. Our findings that patients with PR-negative, ER-negative, and HER-2 positive status were more likely to achieve pCR align with these results. Liu et al. (28) achieved an AUC of 0.779 using ROC analysis of clinical features through univariate and multivariate analysis. Whereas, our R-model, based on the GBM algorithm, yielded an AUC of 0.807, indicating superior predictive performance, likely due to the advantages of ML. A study (29) suggests that radiomics models, which capture tumor size and heterogeneity, often outperform clinical models. Ultrasonography examination, with its wide range of availability, lower expenditure, live properties, noninvasive nature, and outstanding resolution of soft tissue, provides a significant advantage in capturing detailed structural information (30). While various studies have explored radiomics models for predicting tumor response to NAC, performance and quality have varied (31). Features extracted at multiple points, with AUC ranging from 0.86 (32) to 0.94 (33), often require multiple patient tests (before treatment, early treatment [after completing two (28) or four NAC cycles] (34), and after treatment), which can be burdensome for patients and clinicians. Our model, which uses only pre-treatment ultrasound RFs, achieved an AUC of 0.851, outperforming the C-model. The optimal features in our radiomics model include GLDM, first-order, GLRLM, GLCM, and SHAP, with GLCM features being the most prevalent. Research shows that GLCM features reflect tumor image changes and inhomogeneity by calculating the relative distance between specific pixels and the correlation coefficient of (35–38) grayscale values in various directions. This may contribute to the superior performance of our prediction model.

### 4.2 GBM model and SHAP interpretation of clinical features combined with ultrasound RFs

We developed a GBM model that integrates Rad score with clinical characteristics, and the results indicate that the combined C-R-model outperforms any single model in predicting pCR (**Figures 5G, H**). This model demonstrates higher accuracy, underscoring its applicability and reliability. Previous research has also highlighted the strong predictive capabilities of the GBM model (39). To further elucidate the GBM model, we utilized SHAP, a powerful tool for interpreting ML models. SHAP offers a practical means to visualize the contributions of individual features, thereby enhancing the clinical applicability of the model and bolstering the confidence of clinical doctors in using predictive models (40). SHAP waterfall plot (**Figures 7B, C, E, F**) accurately predicts the pCR for both samples and visualizes the prediction process. Its predictive results were consistent with the pathologic findings. By detailing the weights and impacts of the four key predictive features (Rad score, PR status, ER status, HER-2 status) in our combined model, SHAP addresses the “black box” issue that often complicates the use of complicated models. This markedly improves the clinical valuation of our model and increases the trust of clinical doctors in predictive models.

### 4.3 Limitations

Our research has several limitations. Firstly, being a retrospective single-center study, there is a potential selection bias that may influence our results. For instance, Asian women often have denser breast tissue (41), which might affect the generalizability of the model. Secondly, variations in ultrasound equipment and examination parameters, due to individual differences among patients, may impact the quality and uniformity of the images. Thirdly, our study covers the period from 2014 to 2023, during which the standards for NAC and patient care evolved. Although our analysis focuses on ultrasound images obtained before treatment, variations in NAC responses over time might still affect the performance of our model. Lastly, as a single-center retrospective study, our findings need to be validated through multicenter research to confirm the reliability and applicability of the GBM model.

## 5 Conclusion

In summary, we established and compared three GBM models to predict the pCR of BC undergoing NAC before treatment. These models included clinical characteristics, ultrasound RFs, and a combination of clinical characteristics and ultrasound RFs. Our findings indicate that the C-R-model, which integrates both clinical characteristics and ultrasound RFs, has the best predictive performance for pCR. SHAP analysis provided a clear explanation for the prediction results of the GBM model, revealing that participants with a high Rad score, PR-negative status, ER-negative status, and HER-2-positive status are more likely to achieve pCR. This model offers rewarding prognostic information on the effectiveness of NAC in treating BC and provides a useful reference for formulating individualized therapeutic strategies.

## Data Availability

The original contributions presented in the study are included in the article/**Supplementary Material**. Further inquiries can be directed to the corresponding author.

## Supplementary material

Supplementary Figure 1 Nomogram. **(A)** A visual nomogram was developed using Rad-score combined with PR status, ER status, HER-2 status. ROC curves for the nomogram of the training set **(B)** and the validation set **(C)**.
