# Supplementary figures and images for: Machine learning for predicting neoadjuvant chemotherapy effectiveness using ultrasound radiomics features and routine clinical data of patients with breast cancer
Source: Front Oncol. 2025 Jan 14;14:1485681. doi: 10.3389/fonc.2024.1485681 (PMC11803464; doi:10.3389/fonc.2024.1485681)

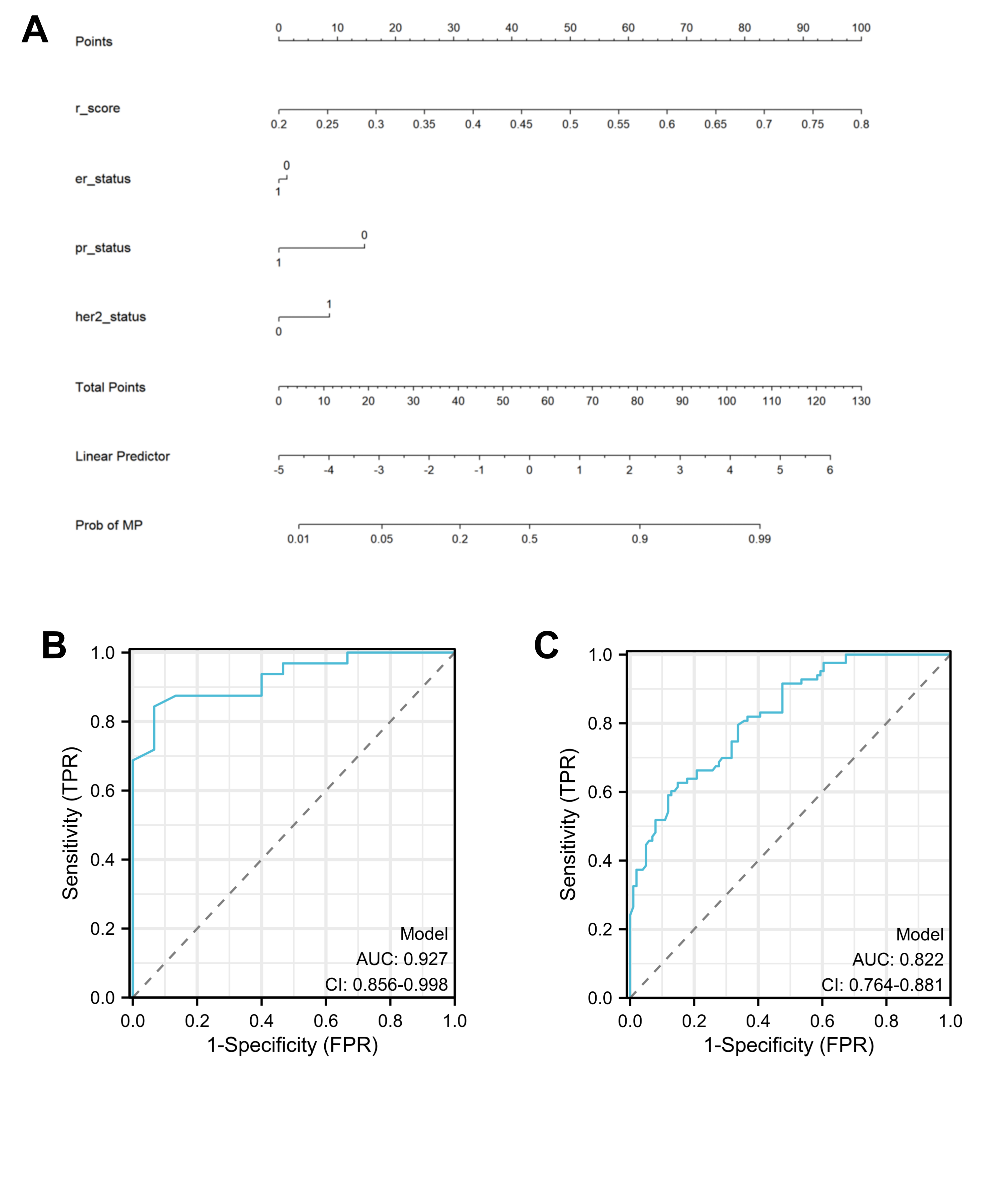

Supplement: Supplementary Figure 1 — Nomogram. (A) A visual nomogram was developed using Rad-score combined with PR status, ER status, HER-2 status. ROC curves for the nomogram of the training set (B) and the validation set (C). [file Image1.jpeg]
